# Supplementary material for: YY1 binding is a gene-intrinsic barrier to Xist-mediated gene silencing
Source: EMBO Rep. 2024 Apr 23;25(5):2258–77. doi: 10.1038/s44319-024-00136-3 (PMC11094009; doi:10.1038/s44319-024-00136-3)
Supplement: Supplementary file 8 — Expanded View Figures [file 44319_2024_136_MOESM8_ESM.pdf]

Expanded View Figures

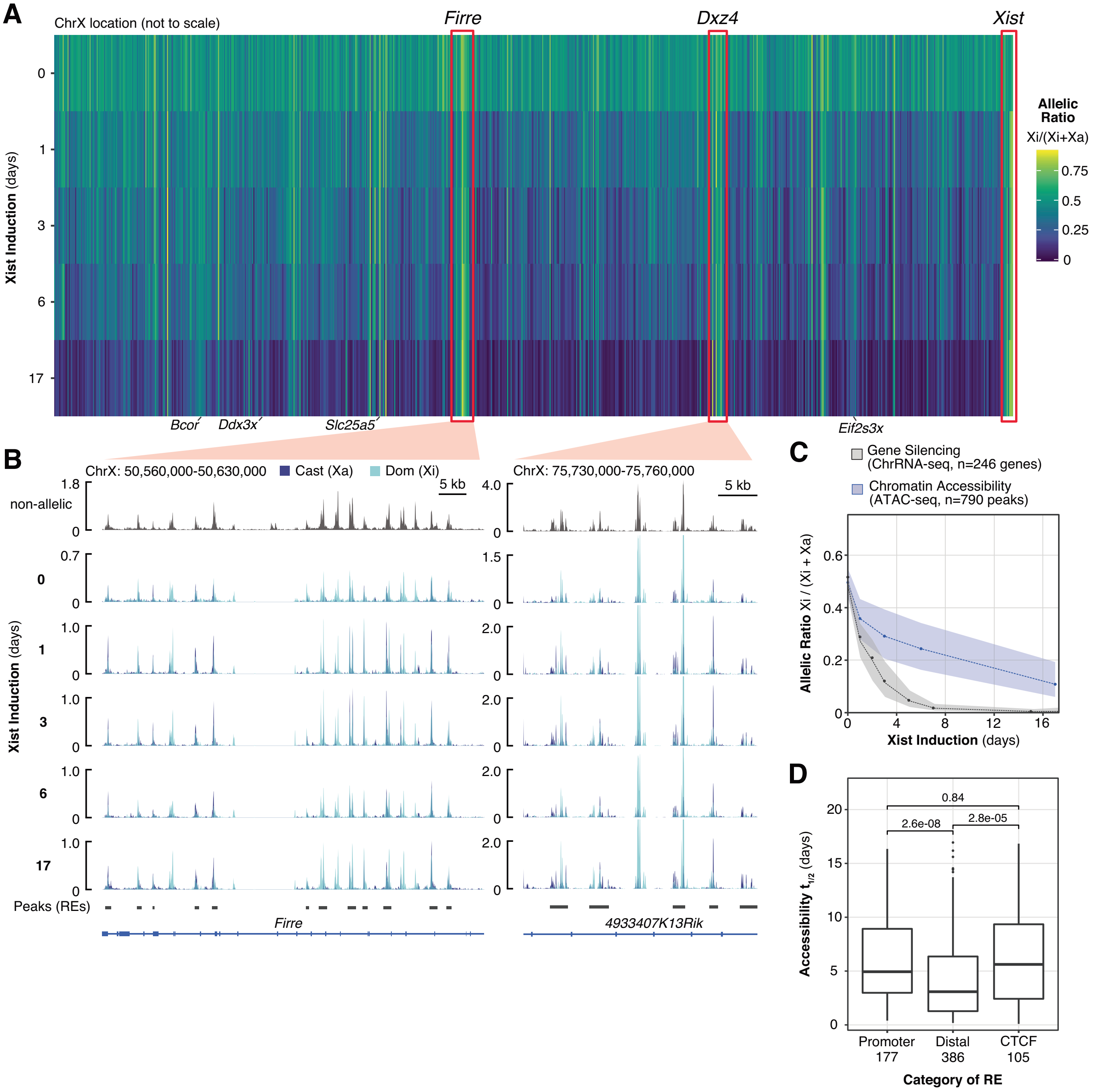

Figure EV1. Allele-specific ATAC-seq over a time course of inducible XCI.

(**A**) Heatmap of allelic ratios for each X-linked RE in iXist-ChrX$_{Dom}$ cells over a time course of Xist induction with NPC differentiation. Three loci containing clusters of RE which increase in accessibility on Xi (*Firre*, *Dxz4* and *Xist*) are indicated by red boxes. Locations of escapee gene loci which retain biallelic accessibility *Ddx3x*, *Slc25a5* and *Eif2s3x* are also labelled. *Bicor* is efficiently silenced but contains a cluster of intragenic REs which remain biallelically accessible. Allelic ratios are the average of 2 or 3 replicates for each timepoint. Note that REs on ChrX distal to *Xist* (i.e., 103–165 Mb) are not amenable to allelic analysis due to a recombination event during derivation of the iXist-ChrX$_{Dom}$ line (Nesterova et al, 2019). (**B**) ATAC-seq genome tracks of *Firre* and *Dxz4* loci over a time course of Xist induction with NPC differentiation. Tracks are the average of duplicates for each timepoint. (**C**) Ribbon plot comparing the dynamics of decreasing RE chromatin accessibility with gene silencing dynamics. Dashed lines connect median averages for each timepoint and shaded areas trace interquartile ranges. (**D**) Boxplots of accessibility halftimes grouped by a categorisation of regulatory elements as promoters (+/-500bp TSS), CTCF sites (CTCF ChIP-seq from GSE144336) or non-CTCF distal elements. Boxes span first to third quartiles with whiskers extending to 1.5 * the interquartile range and a central median line. Significance (*P* values) calculated by Wilcoxon rank-sum test. Numbers of REs in each category are indicated below.

© Crown

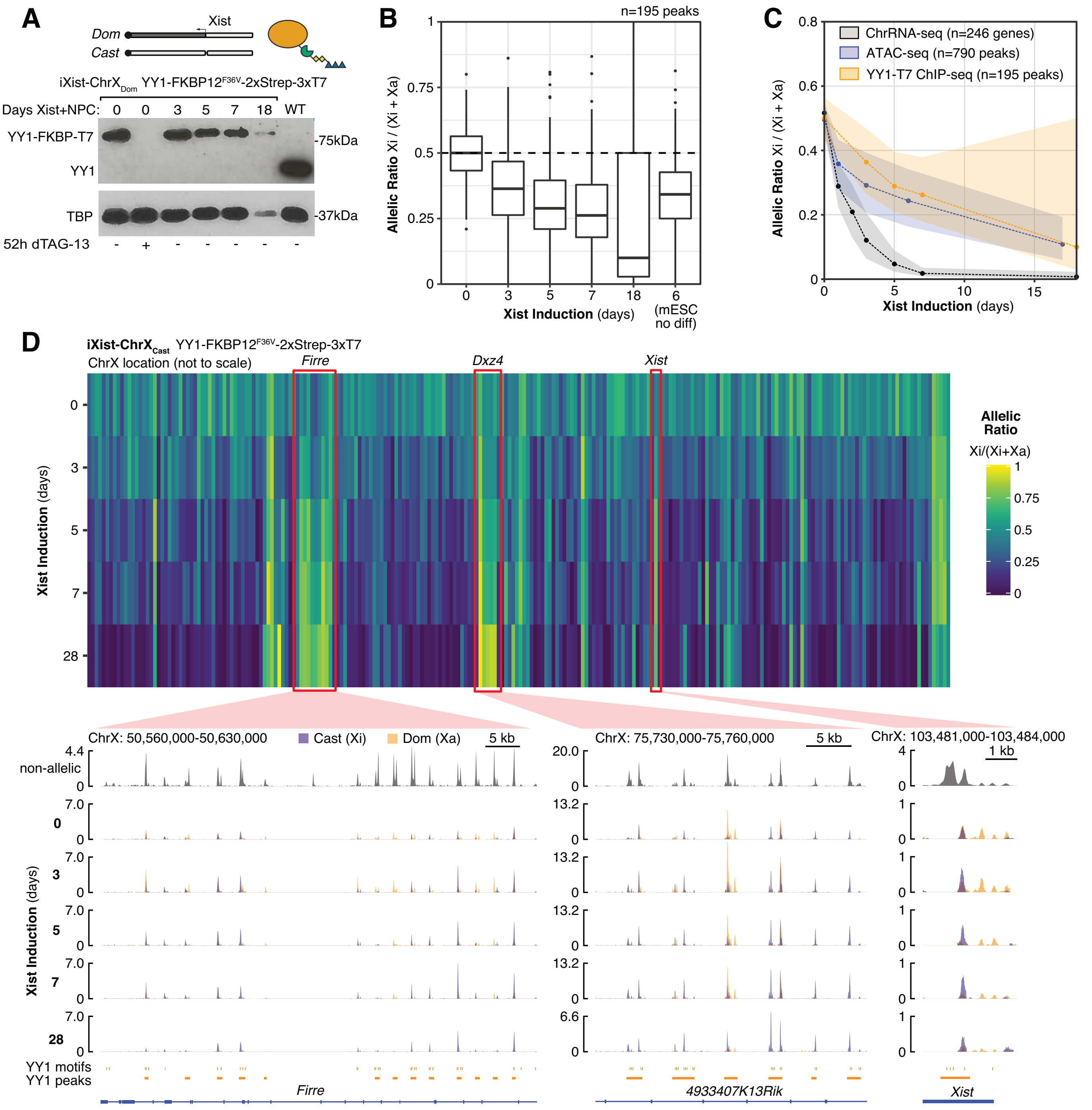

Figure EV2. YY1-T7 ChIP-seq over a time course of inducible XCI.

(**A**) Above: Schematic of the iXist-ChrX$_{Dom}$ YY1-FKBP12^{F36V}-2xStrep-3xT7 cell line. Below: Western blot showing expression of the YY1-degron fusion protein in mESCs and over the course of NPC differentiation, as well as acute degradation upon addition of dTAG-13. TBP acts as a loading control. (**B**) Boxplot of decreasing YY1 binding on Xi, as measured by the allelic ratio in peak regions, over a time course of Xist induction with NPC differentiation (iXist-ChrX$_{Dom}$ background). Boxes span first to third quartiles with a central line indicating the median. Whiskers extend to 1.5 * the interquartile range and outliers outside this range are plotted as separate points. (**C**) Ribbon plot comparing the dynamics of decreasing YY1 binding with decreasing chromatin accessibility and with gene silencing (iXist-ChrX$_{Dom}$ background). Dashed lines connect median averages for each timepoint and shaded areas trace interquartile ranges. (**D**) Above: Heatmap of YY1-T7 ChIP-seq allelic ratios for each X-linked YY1-binding peak over a time course of Xist induction with NPC differentiation (iXist-ChrX$_{Cast}$ background). Below: YY1-T7 genome tracks showing three loci important for the 3D organisation of Xi (*Firre*, *Dxz4* and *Xist*) where Xi-specific YY1 binding increases over the time course of Xist induction.

 © Crown

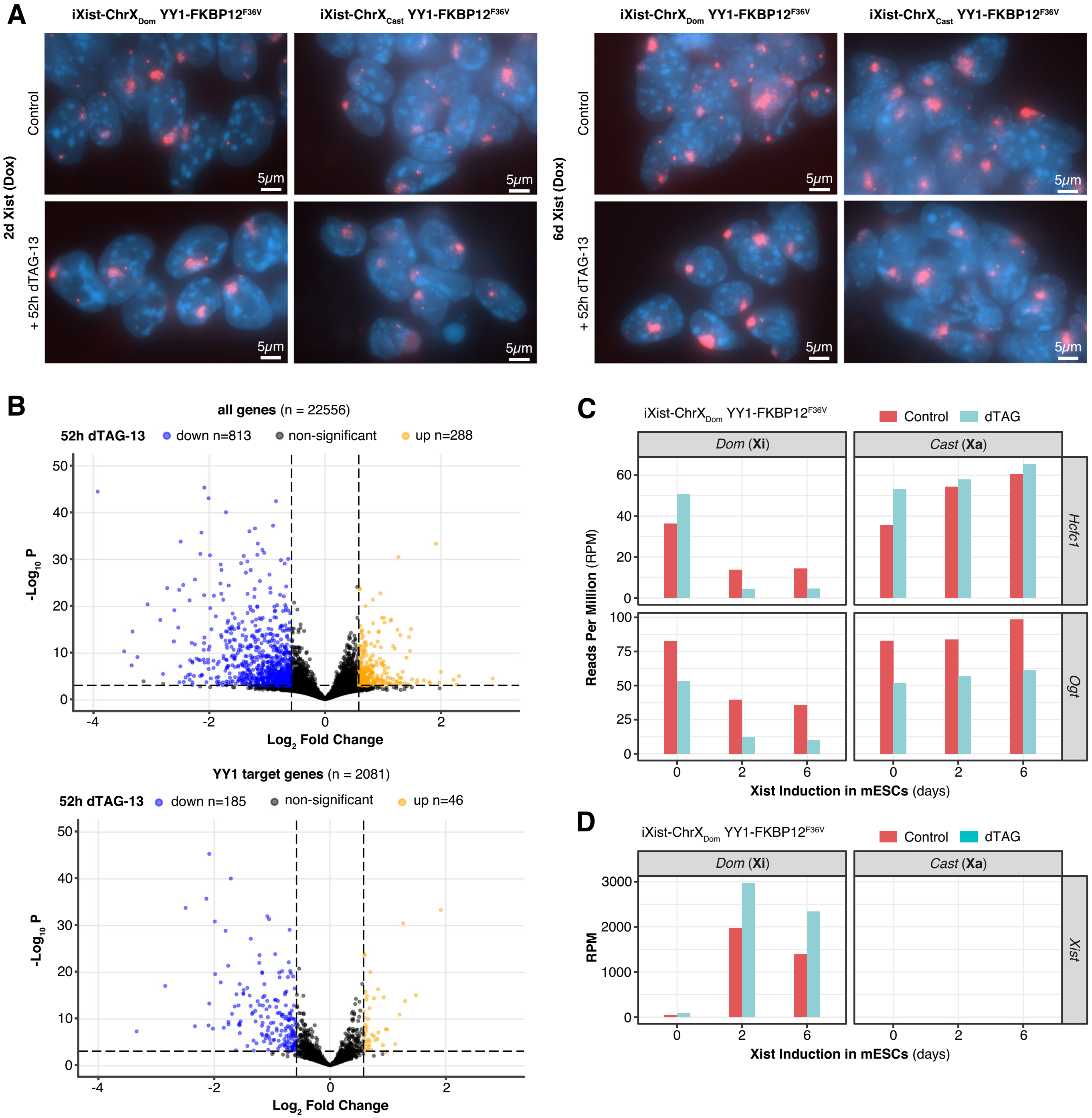

Figure EV3. Xist RNA localisation and gene expression changes upon YY1 degradation.

(**A**) Maximal Z-projection images from Xist RNA-FISH performed on YY1-FKBP12^{F36V}-2xStrep-3xT7 mESCs after 2 days of Xist induction +/− 52 h dTAG-13 (left) or 6 days of Xist induction +/− 52 h dTAG-13 (right). (**B**) Enhanced Volcano plot of genome-wide gene expression changes upon 52 h YY1 degradation. All samples (6 dTAG-treated and 6 control) were included in the analysis regardless of Xist induction status. Thresholds to define strongly differentially expressed genes were set at fold change >1.5 and adjusted *P* value < 0.01 (Wald test with Benjamini–Hochberg correction using DESeq2 (Love et al, 2014)). The lower plot only shows genes defined as direct "YY1 targets" by the presence of a promoter YY1 ChIP-seq peak. (**C**) Relative allelic expression of two example YY1 target genes, *Hcfc1* and *Ogt*, in ChrRNA-seq experiments performed in the iXist-ChrX$_{Dom}$ line. (**D**) Levels of chromatin-associated Xist RNA upon YY1 degradation in ChrRNA-seq experiments performed in the iXist-ChrX$_{Dom}$ line.

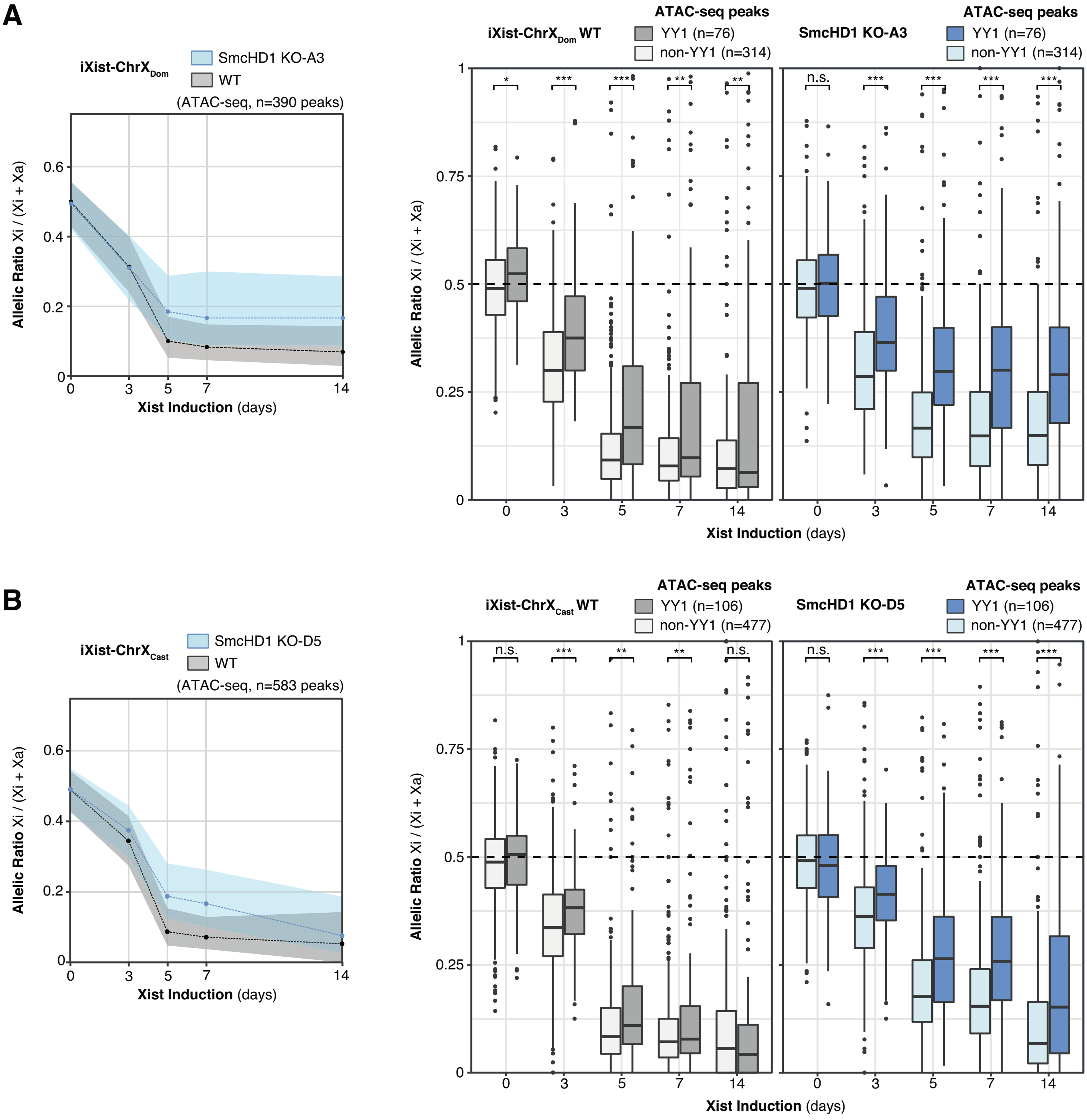

Figure EV4. Allele-specific ATAC-seq of SmcHD1 KO cell lines upon Xist induction with NPC differentiation.

(**A**) Left: Ribbon plot comparing Xi accessibility of ATAC-seq peaks in SmcHD1 KO cell line A3 to wild-type (WT) iXist-ChrX$_{Dom}$. Greater residual Xi accessibility in SmcHD1 KO is evident at later timepoints of Xist induction with NPC differentiation, after the time window of SmcHD1 recruitment to Xi in iXist-ChrX cells (between days 3 and 5). Right: Boxplots comparing allelic ratios of YY1 binding versus non-YY1 REs over the ATAC-seq time courses in iXist-ChrX$_{Dom}$ WT and SmcHD1 KO-A3. Boxes span first to third quartiles with a central line indicating the median. Whiskers extend to 1.5 * the interquartile range and outliers outside this range are plotted as separate points. Significance calculated by Wilcoxon rank-sum test. *, ** and *** indicate P values below, 0.01, 0.001 and 0.0001, respectively. The numbers of YY1-binding and non-YY1 ATAC-seq peaks (REs) which are amenable to allelic analysis are displayed above the plot. (**B**) As (**A**) but for SmcHD1 KO cell line D5 derived from the reciprocal iXist-ChrX$_{Cast}$ line.

 © Crown
